# Impact of the use of nephrotoxic drugs in critically ill pediatric patients

**DOI:** 10.5935/0103-507X.20200093

**Published:** 2020

**Authors:** Jáder Pereira Almeida, Paulo Ramos David João, Lucimary de Castro Sylvestre

**Affiliations:** 1 Pediatric Intensive Care Unit, Hospital Pequeno Príncipe - Curitiba (PR), Brazil.; 2 Department of Pediatric Nephrology, Hospital Pequeno Príncipe - Curitiba (PR), Brazil.

**Keywords:** Acute kidney injury, Toxicity, Drug-related side effects and adverse reactions, Child, Intensive care units, pediatric, Lesão renal aguda, Toxicidade, Efeitos colaterais e reações adversas relacionados a medicamentos, Criança, Unidades de terapia intensiva pediátrica

## Abstract

**Objective:**

To evaluate the association between the use of nephrotoxic drugs and acute kidney injury in critically ill pediatric patients.

**Methods:**

This was a retrospective cohort study involving all children admitted to the intensive care unit of a pediatric hospital during a 1-year period. Acute kidney injury was defined according to the KDIGO classification. Patients with a length of hospital stay longer than 48 hours and an age between 1 month and 14 years were included. Patients with acute or chronic nephropathy, uropathy, congenital or acquired heart disease, chronic use of nephrotoxic drugs, rhabdomyolysis and tumor lysis syndrome were excluded. Patients were classified according to the use of nephrotoxic drugs during their stay at the pediatric intensive care unit.

**Results:**

The sample consisted of 226 children, of whom 37.1% used nephrotoxic drugs, 42.4% developed acute kidney injury, and 7.5% died. The following drugs, when used alone, were associated with acute kidney injury: acyclovir (p < 0.001), vancomycin (p < 0.001), furosemide (p < 0.001) and ganciclovir (p = 0.008). The concomitant use of two or more nephrotoxic drugs was characterized as an independent marker of renal dysfunction (p < 0.001). After discharge from the pediatric intensive care unit, renal function monitoring in the ward was inadequate in 19.8% of cases.

**Conclusion:**

It is necessary for intensivist physicians to have knowledge of the main nephrotoxic drugs to predict, reduce or avoid damage to their patients.

## Abstract (pt)

**Objetivo:**

Avaliar a associação entre uso de drogas nefrotóxicas e lesão renal aguda em pacientes pediátricos graves.

**Métodos:**

Estudo de coorte retrospectivo envolvendo todas as crianças internadas na unidade de terapia intensiva de um hospital pediátrico durante o período de 1 ano. A lesão renal aguda foi definida pela classificação KDIGO. Foram incluídos pacientes com tempo de internação maior que 48 horas e idade entre 1 mês e 14 anos. Foram excluídos aqueles com nefropatia aguda ou crônica, uropatia, cardiopatia congênita ou adquirida, uso crônico de drogas nefrotóxicas, rabdomiólise e síndrome de lise tumoral. Os pacientes foram classificados quanto ao uso de drogas nefrotóxicas durante internação na unidade de terapia intensiva pediátrica.

**Resultados:**

A amostra foi composta por 226 crianças, sendo que 37,1% fizeram uso de drogas nefrotóxicas, 42,4% desenvolveram lesão renal aguda e 7,5% morreram. As medicações que, isoladamente, apresentaram associação com lesão renal aguda foram aciclovir (p < 0,001), vancomicina (p < 0,001), furosemida (p < 0,001) e ganciclovir (p = 0,008). O uso concomitante de duas ou mais drogas nefrotóxicas foi caracterizado como marcador independente de disfunção renal (p < 0,001). Após alta da unidade de terapia intensiva pediátrica, o acompanhamento da função renal na enfermaria foi inadequado em 19,8% dos casos.

**Conclusão:**

É necessário que o médico intensivista tenha conhecimento das principais drogas nefrotóxicas, de modo a prever, reduzir ou evitar danos a seus pacientes.

## INTRODUCTION

Acute kidney injury (AKI) is a complication often found in patients admitted to the pediatric intensive care unit (ICU) and is characterized by a reduced glomerular filtration rate and/or urine output.^(1,2)^ The main complications related to this disorder are increased fluid overload, reduced excretion of nitrogen compounds, and fluid and electrolyte and acid-base disorders. In addition, patients have longer hospital stays and a higher risk of death.^(3,4)^

Drug nephrotoxicity is one of the main causes of AKI in critically ill children.^(5,6)^ There are multiple mechanisms of renal injury, and the most prevalent are those that cause a reduction in the glomerular filtration rate, acute tubular necrosis, acute interstitial nephritis and renal tubular obstruction.^(7)^

Studies in the pediatric ICU show an association between the use of nephrotoxic drugs (NTDs) and AKI. Gupta et al., in a study involving 536 patients, found that 76% of children with AKI used NTDs, and this association was statistically significant (p = 0.007).^(8)^ Similar results were found in the studies by Freire et al. and Bresolin et al., in which 62% and 39% of patients, respectively, who developed AKI used NTDs, with significant results.^(9,10)^ The main NTDs studied were vancomycin, furosemide, aminoglycosides, nonsteroidal anti-inflammatory drugs, amphotericin and antiviral drugs.^(5,6,11,12)^

Despite the importance of the topic in intensive care, few studies have evaluated the incidence and impact of the concomitant use of two or more NTDs in critically ill pediatric patients. McKamy et al. observed that patients who used vancomycin combined with furosemide had a higher risk of AKI than those who used vancomycin alone (p < 0.001).^(13)^ Other studies reinforce this hypothesis.^(14)^

The objective of this study was to evaluate the association between the use of NTDs and AKI in critically ill pediatric patients.

## METHODS

This is a retrospective cohort study conducted with all patients admitted to the pediatric, clinical and surgical ICU of a 22-bed reference pediatric hospital in 2017. The data were extracted from the electronic medical records of the unit. The study was approved by the Research Ethics Committee of the *Hospital Infantil Pequeno Príncipe* (CAAE: 98429818.5.0000.0097), according to resolution 466/12. The free and informed consent term was waived in the study.

The inclusion criteria were length of stay in the pediatric ICU longer than 48 hours and age between 1 month and 14 years. The exclusion criteria were a history of chronic kidney disease, urogenital disease or kidney transplant; presence of AKI on admission to the pediatric ICU; congenital or acquired heart disease; chronic use of NTDs; creatine phosphokinase ≥ 1,000 U/L; leukocytes ≥ 50,000/mm³ and/or admission for induction chemotherapy; readmission to the pediatric ICU during the same hospital stay; a lack of baseline serum creatinine data; and absence of serum creatinine levels during the pediatric ICU stay.

Nephrotoxic drug is defined as a drug that has the potential to cause AKI as an adverse event^(15)^ (Table 1). The sample was screened for the use of NTDs immediately before and during admission to the pediatric ICU for a maximum of 15 days after admission. Once the use of NTDs was identified, renal function was monitored until hospital discharge or death. The duration of NTD use was defined as the number of days the child received this type of drug and was cumulatively determined for each drug used.

**Table 1:** Nephrotoxic drugs

Acyclovir	Gentamicin
Amikacin	Ibuprofen
AmphotericinB	Ifosfamide
Captopril	Lithium
Carboplatin	Mesalazine
Cyclosporine	Methotrexate
Cidofovir	Sirolimus
Cisplatin	Sulfasalazine
Dapsone	Tacrolimus
Enalapril	Tobramycin
Foscarnet	Topiramate
Furosemide	Trometamol
Ganciclovir	Vancomycin

Acute kidney injury was defined according to the Kidney Disease: Improving Global Outcomes (KDIGO) classification.^(1)^ Patients with a creatinine (Cr) increase < 0.3mg/dL or < 1.5-fold compared to the baseline serum level were classified as without AKI; those with a Cr increase ≥ 0.3mg/dL or between 1.5- and 1.9-fold compared to baseline Cr were classified as stage 1; those with a 2- to 2.9-fold increase in Cr were classified as stage 2; and those with a ≥ 3-fold increase in Cr were classified as stage 3. In this study, urine output was not used to classify the patient as having AKI because drug nephrotoxicity is characterized as nonoliguric.^(15)^ The baseline Cr was the Cr value within the normal range that the patient had in the 3 months before admission to the pediatric ICU according to the unit’s electronic database.

The variables of interest included age, sex, reason for admission, length of hospital stay, Pediatric Risk of Mortality 2 (PRISM 2) score, vasoactive drug use (VAD), presence of AKI, AKI stages, timepoint of the stay at which AKI developed, use of NTDs alone or in combination, duration of NTD use, time between the use of NTDs and the onset of AKI and time between AKI and normalization of renal function or death.

For statistical analysis, the software Epi Info ^TM^ Windows 7.2 and Microsoft® Office Excel 2017 were used. For qualitative variables, frequencies and proportions were calculated. For quantitative variables, we evaluated the mean, standard deviation, median, quartiles and maximum and minimum values. Bivariate analyses were performed using the chi-square test, Fisher’s exact test and Pearson’s correlation coefficient (r), with the calculation of odds ratio (ORs) and 95% confidence intervals (95%CIs) a significance level of 5% (p ≤ 0.05). Logistic regression was used to rule out possible confounders in the association between the use of NTDs and AKI.

## RESULTS

The population admitted to the pediatric ICU during the study period comprised 710 patients; 484 children were excluded, resulting in a sample of 226 patients. Among the excluded patients, 219 patients had a length of stay of less than 48 hours; 77 had AKI on admission; 69 were older than 14 years; 46 had congenital or acquired heart disease; 21 presented with uropathy; 19 had chronic kidney disease; 19 did not have all the data necessary to calculate the variables of interest; nine chronically used NTDs; three were kidney transplant recipients; one patient had rhabdomyolysis; and one had a risk of tumor lysis syndrome.

The sample was predominantly male (54.4%), with a mean age of 3.5 years and a mean baseline Cr of 0.24mg/dL. Hemodynamic shock was the reason for hospitalization in 10.2% (23) of patients, and VAD was used in 17.6% (40) of the sample. The mean length of stay in the pediatric ICU was 10 days, with a mean expected mortality rate at admission of 3% according to PRISM 2, and 7.5% (17) progressed to death (Tables 2 and 3).

**Table 2:** Clinical profile of the sample studied

Variables	
Sex male	123 (54.4)
Age (years)	2 (0 - 6)
Baseline creatinine	0.24 ± 0.08
Reason for admission	
Respiratory failure	84 (37.2)
Monitoring	78 (34.5)
Reduced level of consciousness	41 (18.1)
Hemodynamic shock	23 (10.2)
PRISM 2	1.5 (0.8 - 3.4)
Use of vasoactive drugs	40 (17.6)
Use of NTDs	84 (37.1)
AKI	96 (42.4)
Stage 1	55 (57.2)
Stage 2	27 (28.2)
Stage 3	14 (14.6)
Duration of NTD use (days)	10 (4 - 14)
Time between NTD and the onset AKI (days)	2 (1 - 5)
Time between AKI and creatinine normalization (days)	2 (1 - 6.5)
Length of stay in the pediatric ICU (days)	6 (4 - 11)
Death	17 (7.5)

PRISM 2 - Pediatric Risk of Mortality 2; AKI - acute kidney injury; NTD - nephrotoxic drug; ICU - intensive care unit. Results are expressed as n (%), median (p25% - p75%) or mean ± standard deviation.

**Table 3:** Comparison of patients regarding the use of nephrotoxic drugs

Variable	NTDs		Total	OR	95%CI	p value
	Yes	No				
Age						
Infant	38 (45.3)	59 (41.6)	97	1	-	-
Preschool age	23 (27.4)	37 (26)	60	1.00	0.52 - 1.95	0.98
School age	11 (13.1)	32 (22.5)	43	0.56	0.25 - 1.24	0.15
Adolescent	12 (14.2)	14 (9.9)	26	1.39	0.58 - 3.32	0.46
Sex						
Male	42 (50)	81 (57)	123	0.75	0.43 - 1.29	0.3
Female	42 (50)	61 (43)	103	1	-	-
Reason for admission						
Monitoring	28 (33.3)	50 (35.3)	78	1	-	-
Respiratory failure	27 (32.1)	57 (40)	84	0.89	0.46 - 1.72	0.74
Hemodynamic shock	14 (16.7)	9 (6.4)	23	2.91	1.12 - 7.60	0.02
Decreased consciousness	15 (17.9)	26 (18.3)	41	1.08	0.49 - 2.39	0.83
PRISM 2						
≥ 10%	6 (7.1)	5 (3.5)	11	2.1	0.62 - 7.13	0.22
< 10%	78 (92.9)	137 (96.5)	215	1	-	-
Vasoactive drug						
Yes	29 (34.5)	11 (7.7)	40	6.27	2.93 - 13.45	< 0.001
No	55 (65.5)	131 (92.3)	186	1	-	-
Length of stay (days)						
≥ 7	52 (62)	54 (38)	106	2.64	1.51 - 4.61	< 0.001
< 7	32 (38)	88 (62)	120	1	-	-
Death						
Yes	12 (14.3)	5 (3.5)	17	4.56	1.54 - 13.46	0.003
No	72 (85.7)	137 (96.5)	209	1	-	-
Total	84	142	226			

NTDs - nephrotoxic drugs; OR - odds ratio; 95%CI - 95% confidence interval; PRISM 2 - Pediatric Risk of Mortality 2. Results are expressed as n (%) when not otherwise indicated.

Among the subjects, 42% (96) developed AKI. Of these, 57.2% (55) had stage 1 disease, 28.2% (27) had stage 2 disease, and 14.6% (14) had stage 3 disease. The variables that were associated with renal dysfunction were ICU admission due to hemodynamic shock (p < 0.001), PRISM 2 ≥ 10% (p = 0.037), use of VAD (p < 0.001) and use of NTDs alone or in combination (p < 0.001) (Table 4).

**Table 4:** Association of clinical and demographic variables with acute kidney injury in critically ill pediatric patients

Variable	AKI		Total	OR	95%CI	p value
	Yes	No				
Age						
Infant	50 (51.5)	47 (48.5)	97	1	-	-
Preschool-age	26 (43.3)	34 (56.7)	60	1.17	0.52 - 2.66	0.69
School-age	12 (28)	31 (72)	43	1.24	0.50 - 3.06	0.63
Adolescent	8 (30.7)	18 (69.3)	26	0.61	0.17 - 2.25	0.46
Sex						
Male	50 (40.6)	73 (59.4)	123	0.84	0.49 - 1.44	0.54
Female	46 (44.6)	57 (55.4)	103	1	-	-
Reason for admission						
Monitoring	26 (33.3)	52 (66.7)	78	1	-	-
Respiratory failure	32 (38)	52 (62)	84	1.32	0.5 - 3.46	0.57
Hemodynamic shock	16 (69.5)	7 (30.5)	23	9.48	3.16 - 28	< 0.001
Decreased consciousness	22 (53.6)	19 (46.4)	41	2.80	1.01 - 7.76	0.046
PRISM 2						
≥ 10%	8 (72.7)	3 (27.3)	11	3.84	1 - 14.91	0.037
< 10%	88 (41))	127 (59)	215	1	-	-
Vasoactive drug						
Yes	31 (77.5)	9 (22.5)	40	6.41	2.87 - 14.28	< 0.001
No	65 (35)	121 (65)	186	1	-	-
Use ≥ 1 DNT						
Yes	57 (67.8)	27 (32.2)	84	5.57	3.07 - 10.03	< 0.001
No	39 (27.5)	103 (72.5)	142	1	-	-
Use ≥ 2 DNT						
Yes	21 (95.5)	1 (4.5)	22	36.12	4.76 - 273.98	< 0.001
No	75 (36.7)	129 (63.3)	204	1	-	-
Total	96	130	226			

AKI - acute kidney injury; OR - odds ratio; 95% CI - confidence interval; PRISM 2 - Pediatric Risk of Mortality 2; NTDs - nephrotoxic drugs. The results expressed as n (%) when not otherwise indicated.

A total of 37.1% (84) of the sample used one or more NTDs. Of these, 73.8% (62) used only one NTD, 21.5% (18) used two combined NTDs and 4.7% (4) used three NTDs, so that these medications were prescribed 110 times among patients in the sample. In decreasing order of frequency, 18.5% (42) of the patients used furosemide, 14.1% (32) used vancomycin, 7% (16) used acyclovir, 2.6% (6) used nonsteroidal anti-inflammatory drugs, 2.2% (5) used ganciclovir, 2.2% (5) used gentamicin, and 1.7% (4) used amikacin (Table 5). The mean number of days between the use of NTD and the onset of AKI was 3.6 days. The drugs that were associated with AKI when used alone were acyclovir (p < 0.001), vancomycin (p < 0.001), ganciclovir (p = 0.008) and furosemide (p < 0.001). When we analyzed the association of these drugs with AKI by means of logistic regression adjusted for admission due to hemodynamic shock, PRISM 2 ≥ 10% and VAD use, the only drug that was identified as an independent marker of AKI was acyclovir (OR = 15.3; 95%CI 4.1 - 57; p < 0.001).

**Table 5:** Analysis of the duration of use of nephrotoxic drugs in the sample studied

NTD	Patients (N)	Mean (days)	Median (days)	SD (days)	Minimum (days)	Maximum (days)
Acyclovir	16	11.4	14	5.1	2	21
Amikacin	4	9	8.5	-	5	14
Anti-inflammatory*	6	2.8	2.5	-	2	5
Furosemide	42	6	4	5.2	2	30
Ganciclovir	5	13.8	14	-	3	24
Gentamicin	5	4	3	-	2	7
Vancomycin	32	11.1	10	7.2	2	40

NTD - nephrotoxic drug; SD - standard deviation.

* Includes ibuprofen and trometamol.

The most commonly used combination was furosemide + vancomycin (13), followed by furosemide + acyclovir (4). When we analyzed the association of the use of two or more NTDs with AKI by means of logistic regression adjusted for admission due to hemodynamic shock, PRISM 2 ≥ 10% and VAD use, we found that using ≥ 2 NTDs concomitantly was an independent marker of AKI (p < 0.001) (Table 6). In addition, hemodynamic shock (OR = 3.6; 95%CI 1.03 - 13; p = 0.04) and VAD use (OR = 12.8; 95%CI 5.1 - 31.8; p < 0.001) were also classified as independent AKI markers.

**Table 6:** Multivariate logistic regression for the adjustment of possible confounders in the association between concomitant use of nephrotoxic drugs and acute kidney injury

≥ 2 NTDs	AKI OR (95%CI)
Stages 1 + 2 + 3 (reference group: without AKI)	
Not adjusted	36.1 (4.7 - 272.9)
Adjusted - admission due to shock	37 (4.8 - 281.6)
Adjusted - PRISM 2 ≥ 10%	33 (4.3 - 251.6)
Adjusted - VAD	25.1 (3.2 - 194.7)
Adjusted - shock, PRISM 2 and VAD	27.5 (3.5 - 215.5)
Stages 2 + 3 (reference group: stage 1 + without AKI)	
Not adjusted	11.4 (4.4 - 29.1)
Adjusted - admission due to shock	13.5 (4.9 - 37.1)
Adjusted - PRISM 2 ≥ 10%	10 (3.7 - 27)
Adjusted - VAD	6.9 (2.1 - 22)
Adjusted - shock, PRISM 2 and VAD	7.8 (2.4 - 25.9)

NTDs - nephrotoxic drugs; AKI - acute kidney injury; OR - odds ratio; 95%CI - 95% confidence interval; PRISM 2 - Pediatric Risk of Mortality 2; VAD - vasoactive drug.

The mean duration of NTD use in patients without AKI was 8 days (± 4.9). Patients with stage 1 AKI had a mean duration of use of 12 days (± 12.2), and those with stage 2 or 3 AKI had a mean duration of use of 13.1 days (± 8.8). The correlation analysis between the time of use of NTD and AKI stage showed r = 0.21 (p = 0.83).

Regarding renal function recovery time, patients who developed stage 1 AKI required a mean of 3.1 days (± 3.6) for Cr to normalize; patients with stage 2 AKI recovered renal function in 5.4 days (± 4.5), and children with stage 3 recovered renal function in 13.5 days (± 6.6). All patients who were discharged from the ICU showed complete recovery from the condition, but 19.8% of the cases did not adequately monitor renal function in the ward.

Among the patients who died, 5.9% (1) were in the group without AKI, 11.8% (2) had stage 1 AKI, 29.4% (5) had stage 2 AKI, and 52.9% (9) were classified as stage 3, and this result was statistically significant (p < 0.001).

## DISCUSSION

Drug nephrotoxicity is one of the main causes of AKI in pediatric ICUs.^(6)^ However, it is difficult to assess its real impact on the genesis of the problem due to the multifactorial nature of renal dysfunction in critically ill children, especially in those with hemodynamic instability.^(16,17)^ To reduce the risk of bias in the association between NTDs and AKI, this study used stricter inclusion and exclusion criteria than previous studies on the subject for the selection of the sample.^(18-20)^

After adjustment by logistic regression, the only NTD identified as an independent marker of AKI was acyclovir, and it was used mainly for empirical treatment of meningoencephalitis caused by herpesvirus type 1. Its mechanism of injury is characterized by the formation of crystals and the obstruction of tubules, and the risk of renal dysfunction is dose dependent.^(21,22)^ Slater et al. found that ganciclovir, furosemide and gentamicin were the main drugs associated with AKI in critically ill patients (p < 0.05).^(6)^ The different methodological profiles used for sample selection may explain the divergence between the results, and additional, preferably prospective, studies are needed to identify the main NTDs associated with renal dysfunction in critically ill children.

When we analyzed the concomitant use of these drugs, the risk of AKI was observed to increase progressively as the number of NTDs used increased.^(8,14,16,23,24)^ McKamy et al., in a retrospective study conducted in a pediatric ICU, identified a 3- to 9-fold increased risk of AKI when vancomycin was used concomitantly with furosemide (p < 0.05).^(13)^ Our study showed similar results, and this variable was characterized as an independent marker of AKI (p < 0.001). This finding is relevant in the context of critically ill children because drug-induced renal dysfunction is potentially reversible.^(2)^

In addition, studies show that after recovery from AKI, the patient has an increased risk of chronic kidney disease as a result of the development of proteinuria and hypertension and a reduced glomerular filtration rate.^(25,26)^ Therefore, prevention measures are essential to avoid short- and long-term complications, and drugs with fewer adverse effects should always be chosen when possible. Special attention should be given to critically ill children with heart disease, cancer, nephrotic syndrome and those in the neonatal period as they are usually exposed to large amounts of NTDs.^(27,28)^

We observed that one-fifth of the children using NTDs had inadequate monitoring of renal function after discharge to the ward. Other studies show that up to 33% of patients with AKI are inadequately managed.^(1)^ Thus, we emphasize that serially assessing renal function for early identification of the problem, avoiding concomitant use of NTDs, adjusting the dose of drugs according to the estimated glomerular filtration rate and maintaining a normovolemic state are imperative measures in the management of these patients.^(7)^

Our study had some limitations intrinsic to retrospective studies. Approximately 2.6% of the study population was excluded due to the lack of data needed to calculate the variables of interest, which may impact the result. The sample size is considered small compared to other studies in the literature; consequently, there is a greater risk of type 2 random error in the analysis of drug classes that were rarely used by patients, such as nonsteroidal anti-inflammatory drugs and aminoglycosides.^(12,26)^ Although radiocontrast agents used for imaging are considered nephrotoxic, it was not possible to identify the different types of agents and their respective osmolarities in this study, and thus, they were not included in the list of NTDs. The first author was responsible for data collection, and the use of a structured questionnaire with a systematic data storage methodology minimized the risk of bias. Finally, we chose to analyze a small group of drugs that includes the drugs that were most commonly used in the unit where the study was conducted.

## CONCLUSION

Acyclovir alone was the drug most strongly associated with renal dysfunction in this study. In addition, concomitant use of nephrotoxic drugs was characterized as an independent marker of acute kidney injury.

To better characterize the impact of the use of nephrotoxic drugs in critically ill children, further studies on the subject are needed, and the multifactorial genesis of acute kidney injury should be considered.

It is necessary for pediatric intensivists to have knowledge of the main nephrotoxic drugs to predict, reduce or avoid damage to their patients.
